# Lactylation: An Innovative Approach to Disease Control

**DOI:** 10.14336/AD.2024.0918

**Published:** 2025-08-24

**Authors:** Xiaoxin Jiang, Yanyan Yang, Xiaolu Li, Tianxiang Li, Tao Yu, Xiuxiu Fu

**Affiliations:** ^1^Department of Cardiac Ultrasound, the Affiliated Hospital of Qingdao University, Qingdao, China.; ^2^Department of Immunology, School of Basic Medicine, Qingdao University, Qingdao, China.; ^3^Institute for Translational Medicine, The Affiliated Hospital of Qingdao University, Qingdao, China.

**Keywords:** lactylation, metabolism, histone, tumor, inflammation

## Abstract

Assessment of tissue microenvironment lactate levels has emerged as a crucial indicator of microcirculation and early organ dysfunction. Lactylation modification, closely associated with lactate concentration, represents a novel post-translational alteration targeting protein lysine residues. Post-translational modifications are chemical changes capable of modulating protein activity and functionality, serving as a rapid mechanism for enhancing proteomic diversity and influencing various life processes. While previous research primarily focused on histone lactylation, recent studies have revealed the occurrence of lactylation on non-histone proteins, exerting significant effects on gene expression and intercellular communication. Lactylation has been implicated in diverse diseases spanning embryonic development, neuronal excitability, inflammatory responses, cardiovascular conditions, tumor progression, invasion, and aging. Hence, lactylation emerges as a pivotal regulator in numerous pathological conditions. This review delves into the mechanisms underlying lactylation and disease pathogenesis, elucidates the multifaceted roles of lactylation in disease progression, and identifies novel therapeutic targets related to lactylation, offering potential avenues for future clinical interventions.

## 1. Introduction

In 1990, Otto Warburg made the initial observation that tumor tissue consumes more glucose than the surrounding normal tissue. It is widely accepted that even in the presence of oxygen, cancer cells tend to convert glucose into lactate as a means of generating energy. The accumulation of lactate, both inside and outside the cells, provides the most direct evidence of aerobic glycolysis [1]. An expanding array of studies indicates that the occurrence of aerobic glycolysis in cancerous cells primarily results from metabolic reprogramming, which consequently impairs the efficacy of mitochondrial oxidative phosphorylation [2, 3]. The presence of lactate in the tissue microenvironment is notable, as it is a characteristic feature of inflammatory diseases and cancers [4]. Nevertheless, there is also substantial evidence that the Warburg effect is involved in various non-oncological diseases such as pulmonary hypertension, pulmonary fibrosis, Alzheimer's disease, heart failure, and atherosclerosis [5-11].

Since the discovery of lactate, it has been recognized as a crucial substance for cellular energy and metabolism. The introduction of the intercellular lactate shuttle theory in the early 1980s completely overturned the understanding of lactate as a metabolic waste product. The recent acceptance of the intercellular lactate shuttle hypothesis, along with the finding that lactate can be generated and utilized even in the presence of adequate oxygen, researchers have completely changed their perception of lactate [12, 13]. The relationship between lactate and disease occurrence, its role as a signaling molecule between cells, and its function as an immunomodulatory molecule have been extensively studied. While lactate acts as a modulator of the immune system during inflammation and is widely recognized as an anti-inflammatory factor, it may also contribute to the exacerbation of inflammation in certain inflammatory conditions [14]. Lactate frequently exacerbates the proliferation and invasion of tumor cells, assisting them in evading immune surveillance. This may be related to the fact that lactate increases the number of CD8^+^T cells [15]. The accumulation of lactate in the tumor microenvironment can also increase the expression of MMPs, which further promotes tumor progression. Additionally, lactate activates the cell surface G protein-coupled receptor (GPR81), which is highly expressed in various tissues and mediates lactate-induced energy metabolism, neuroprotection, inflammation, and other biological processes [16].

In 2019, a research team headed by Yingming Zhao uncovered that lactate, a byproduct of metabolism, serves as a precursor for the lactylation of histone lysine (Kla) [17]. The specific protein where this modification occurs determines the type of modification, which can be categorized as either histone lactylation modification or non-histone lactylation modification. Subsequent studies have shown that lactylation modifications are involved in the development of various diseases, indicating a correlation between disease progression and the accumulation of lactate. Lactylation modifications, encompassing both histone and non-histone types, play pivotal roles in essential cellular functions and pathophysiological conditions, albeit through disparate regulatory pathways. Specifically, histone lactylation predominantly modifies histones such as H3 and H4 and modulates disease processes primarily via the action of histone deacetylases (HDACs) and histone acetyltransferases (HATs), influencing chromatin architecture and functionality. In contrast, non-histone lactylation affects proteins other than histones, including metabolic enzymes, transcriptional regulators, and signal transduction molecules. This form of lactylation exerts its influence on chromatin structure and function through various enzymes such as lactate dehydrogenase (LDH), pyruvate kinase (PK), and glycerate kinase (GK), which are instrumental in maintaining protein stability, mediating protein-protein interactions, and regulating protein activity.

**Figure 1.**
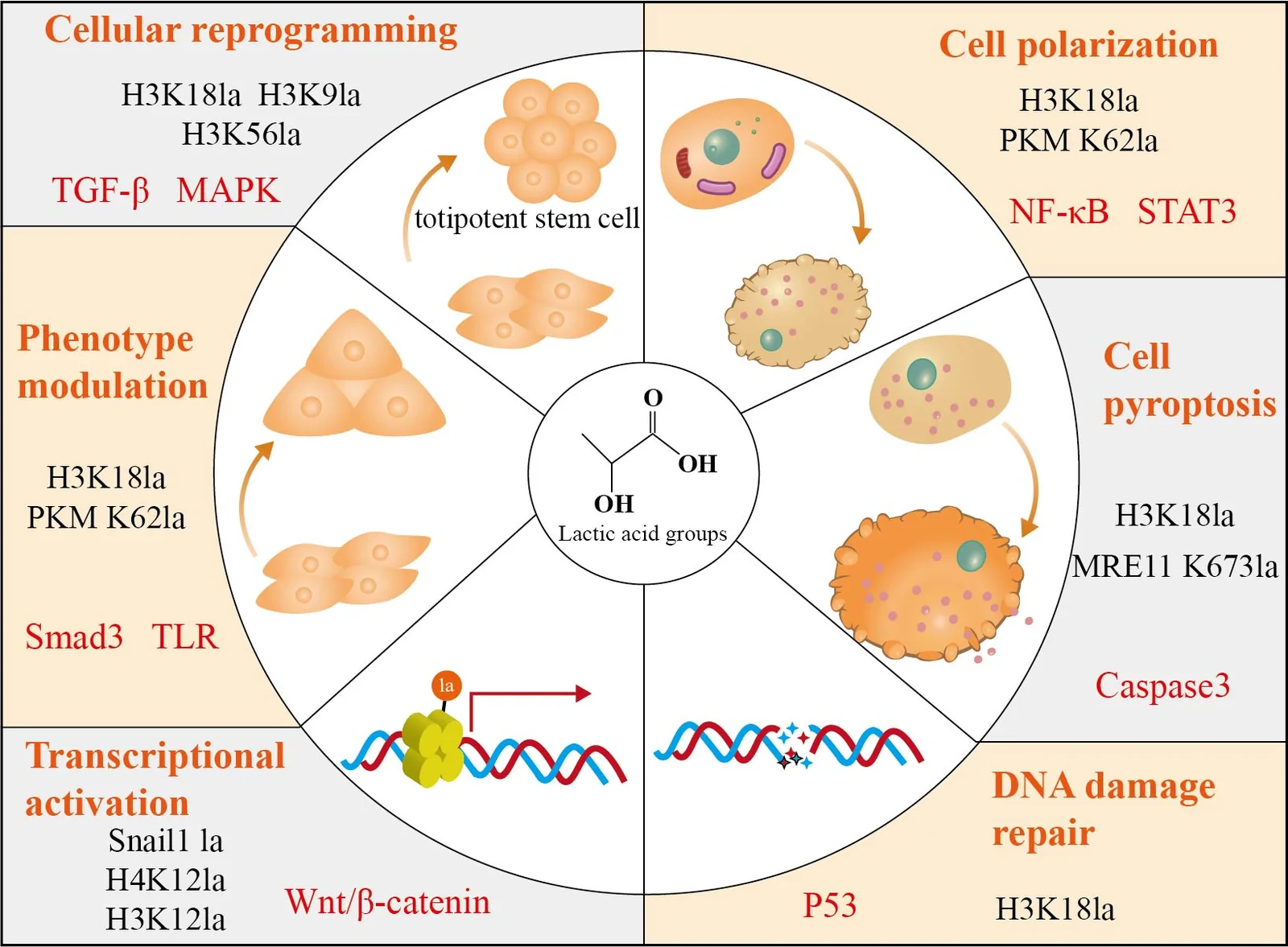
**Cellular functions regulated by lactylation and the corresponding modifications at specific sites**. Histone and non-histone lactylation that mediates cellular functions such as metabolic reprogramming, phenotypic switching, transcriptional activation, cell polarization, cellular focal death, and DNA damage. The red font shows the relevant signaling pathways involved in lactylation. PKM: pyruvate kinase M2; MRE11: meiotic recombination 11.

Similar to methylation and acetylation modifications, histone lactylation modifications modulates gene expression. They play a role in maintaining the balance of bacterially infected M1 macrophages, regulating glucose metabolism in microglia, modulating secretion-associated phenotypes in senescent microglia, mediating immunosuppression and immune escape in tumor cells, as well as facilitating endothelial-to-mesenchymal transition in cardiac myocytes (Fig. 1) [17-21]. Non-histone lactylation also has a critical function in various diseases. For instance, it reduces the expression of pro-inflammatory factors in macrophages and inhibits macrophage polarization. Lactylation occurring on PKM2 can also promote the transition of macrophages to a reparative phenotype [22]. Lactylation of non-histone proteins plays a critical role in oncogenesis through modulation of metabolic pathways including the Krebs cycle and Wnt signaling [23, 24]. A decline in lactylation at the α-myosin heavy chain (α-MHC) K1897 site impairs the interaction between α-MHC and Titin, culminating in myocardial structural damage and consequent cardiac failure. Furthermore, this post-translational modification affects the advancement of various diseases by regulating essential signaling pathways, including the transforming growth factor-beta (TGF-β)/Smad2 and mitogen-activated protein kinase (MAPK) pathways [25-27]. Although non-histone modifications are also involved in critical cellular physiological or pathological processes, there have been relatively fewer studies conducted on this topic, and further in-depth research is needed.

In this comprehensive review, we provide an in-depth examination of the process of lysine lactylation modification, including its distinctive features and the enzymes responsible for its regulation. Additionally, we summarize the regulatory role of lactylation from a pathological perspective, as well as the critical signaling pathways associated with this modification at the cellular level. Lastly, we explore the current challenges faced in lactylation studies and propose potential therapeutic targets for further investigation.

**Table 1:** The effect of lactylation in various diseases.

Classification	Disease	Cell line	Lactylation site	Mechanism	Ref.
**Inflammation**	Ulcerative colitis	Macrophage		TLR signaling adaptor BCAP enhances glycolysis and promotes lactfication by inhibiting FOXO1 and GSK3β; regulating histone modification affects damage repair gene expression and repair macrophage transformation.	[32]
			H3K18	Lactate-producing Saccharomyces cerevisiae inhibits IL-1 β, NLRP 3, and M1 macrophage polarization.	[38]
	Intestinal inflammation	TH17 cells	H3K18	Upregulation of Foxp 3 by lactate inhibition of IL-17A.	[47]
	Sepsis	Macrophage		Kla of HMGB.	[37]
	septic shock			H3K18la-mediated anti-inflammatory effects promote repair gene expression.	[52]
	Pulmonary fibrosis	Macrophage, Pulmonary myofibroblasts		Kla upregulates pro-fibrosis genes Arg1, Opn, Pdgfa, Thbs and Vegfa.	[39]
	Autoimmune Uveitis	Microglial	YY1 K183	YY1 lactylation intensifies autoimmune uveitis by facilitating the transcription of inflammatory mediators that influence microglial activity, thereby worsening the condition.	[50]
	Sepsis-Associated Acute Kidney Injury	Renal proximal tubular epithelial cells	Ezrin-K263	Histone lactylation exacerbates renal impairment by enhancing inflammation, oxidative stress, and apoptosis through the RhoA/ROCK/Ezrin signaling pathway.	[57]
		Macrophage	K62	PKM K62la promotes the transition of macrophages to a repair phenotype.	[22]
**Tumour**	Ocular melanoma	Ocular melanoma cells	H3K18	H3K18la Upgrade YTHDF2; Downgrade PER1/TP53.	[36]
	Cervical cancer	C33A, PHK, MEF cell	G6PD K45	HPV16 E6 affects cervical cancer progression by regulating G6PD K45 lactylation	[63]
	Non-small cell lung cancer	NSCLC cell	H4K8	Promote the expression of SDHA, IDH3G and HIF1-α, inhibit the expression of HK-1, G6PD and PKM.	[24]
	Prostate cancer	Prostate cancer cell	HIF-1α	HIF-1α lactylation promotes the transcription of KIAA1199, facilitating angiogenesis and advancing the progression of prostate cancer.	[72]
	Pancreatic adenocarcinoma	Pancreatic adenocarcinoma cell	NMAAT1	Lactylation of NMNAT1 sustains the nuclear NAD+ salvage pathway, promoting the survival of pancreatic adenocarcinoma cells.	[64]
	Clear cell renal cell carcinoma	786-O cell, A498 cell		VHL forms an oncogenic positive feedback loop with PDGFRβ and ccRCC via histone lactylation.	[40]
	Hepatocellular carcinoma	Hepatocellular carcinoma stem cell	H3K9, H3K56	DML affects histone lactylation by modulating lactate content.	[59]
	Hepatocellular carcinoma	Huh cell		SIRT3 reduction and elevated lactylation of cell cycle protein E2.	[33]
	Colon cancer	Myeloid cell	H3K18la	The lactylation-driven Mettl3-mediated modification of RNA m6A promotes immunosuppression in tumour-infiltrating myeloid cells.	[19]
	Colorectal cancer	CRC cells		The Wnt pathway is activated by β-catenin lactylation.	[23]
	Gastric cancer	Gastric cancer cell			[71]
		Treg cell		Lactate modulates Treg cell production via lactylation of Lys72 in MOESIN, thereby improving MOESIN interaction with TGF-β receptors I and downstream SMAD3 signaling.	[27]
**Cardiovascular disease**	Cerebral ischaemia			Lactylation regulate Ca^2+^ signaling pathway key proteins SLC25A4 (K245) and SLC25A5 (K96).	[26]
	Cerebral infarction	PC12 cell		Reduced LCP1 lactylation.	[42]
	Myocardial infarction	Endothelial cell		Lactate promotes nuclear translocation of Snail1 after hypoxia/MI and lactylation contributes to EndoMT.	[20]
	Vascular calcification	Vascular smooth muscle cells	H3K18	NR4A3 regulates vascular calcification through mediated histone lactylation	[96]
	Atherosclerosis		H4K12	TRAP1 intensifies atherosclerosis by promoting the transcriptional activation of the H4K12la phenotype associated with senescence in smooth muscle cells.	[97]
	Brain ischemic stroke	Astrocyte	ARF1 K73	LRP1 regulates astrocyte mitochondrial translocation and ARF1 K73 lactylation antagonizes postischemic nerve injury	[85]
	Cerebral ischaemia reperfusion	N2a cell	H3K18	LDHA mediated histone lactylation can interact with HMGB1 to induce focal death in CI/R injury.	[41]
	Myocardial infarction	BMDM	H3K18	GCN5 mediates up-regulation of H3K18la; Initiates expression of repair genes Lrg1, Vegf-α and IL-10.	[91]
	Heart failure	H9C2	α-MHC K1897	α-MHC K1897 lactylation regulate the interaction between α-MHC and Titin.	[25]
**Neuropsychiatric disease**	Depression			Stress-induced neuronal excitation induces lactylation.	[100]
	Alzheimer's disease	Microglia	H4K18, H4K5, H4K8, H3K18, H3K23	“Glycolysis-histone lactylation-PKM2” positive feedback regulatory loop.	[18]
**Embryonic**	Pre-implantation embryonic development	Oocyte	H3K23, H3K18	H3K23la, H3K18la and pan Kla in mouse oocytes and preimplantation embryos impair the developmental potential of mouse preimplantation embryos.	[103]
	Endometrial Tolerance		H3K18	Important role of lactate in inducing endometrial H3K18 lactylation and in regulating redox homeostasis and apoptotic balance to ensure successful implantation.	[104]
**Aging**	Brain aging	Senescent microglial cells	H3K18	H3K18la regulates senescence-associated secretory phenotype components IL-6 and IL-8 through activation of the NF-κB signaling pathway regulates senescence-associated inflammation leading to brain aging	[21]
	Intervertebral disk degeneration	Nucleus pulposus cell	AMPKα	Glutamine modulates AMPKα lactylation to delay aging and promote autophagy	[114]

BCAP: B-cell adapter for PI3K; FOXO1: forkhead box protein O1; GSK3β: glycogen synthase kinase 3β: VHL: inactive von Hippel-Lindau; ccRCC: clear cell renal cell carcinoma; PDGFRβ: platelet-derived growth factor receptor β; HK-1: Hexokinase 1: PKM: pyruvate kinase M; SDHA: succinate dehydrogenase A; IDH3G: isocitrate dehydrogenase 3G; DML: demethyzeylasteral; LCP1: lymphocyte cytosolic protein 1; NR4A3: nuclear receptor subfamily 4 group A member 3; LRP1: Low-density lipoprotein receptor-related protein-1; ARF1: ADP-ribosylation factor 1; TRAP1: tumour necrosis factor receptor-associated protein 1

## 2. Global analysis of lysine lactylation

In the groundbreaking study published in 2019, Zhang *et al*. discovered that histone lysine lactylation modifications are prevalent in both human and mouse cells. Through analytical chemistry and mass spectrometry, Zhang *et al.* demonstrated the widespread occurrence of histone lysine lactylation modifications in both human and murine cells. This process, known as histone lactylation, involves the covalent attachment of lactide groups to histone lysine residues and plays a role in gene transcription and gene expression regulation [17]. Subsequent research has revealed that lactylation is not only limited to histones, but also occurs on non-histone proteins. Gao *et al*. utilized LC-MS/MS analysis and determined that lactylated proteins predominantly reside in the nucleus, mitochondria, and cytoplasm of gray molds [28]. Further investigations conducted by Wan *et al*. confirmed that lactylation also has significant regulatory functions on non-histone substrates [29]. In their research, Wu *et al*. have elucidated that tandem mass spectrometric analysis is crucial in the accurate identification of protein lactylation, revealing a broad spectrum of lysine lactylation post-translational modifications that surpass those found in histones [30]. In addition, the investigation highlights an association between the fluctuations of lactylation modifications and the dysregulation of lactate, which is implicated in the etiology of prevalent chronic pathologies, including inflammatory conditions and tumor [30, 31].

**Figure 2.**
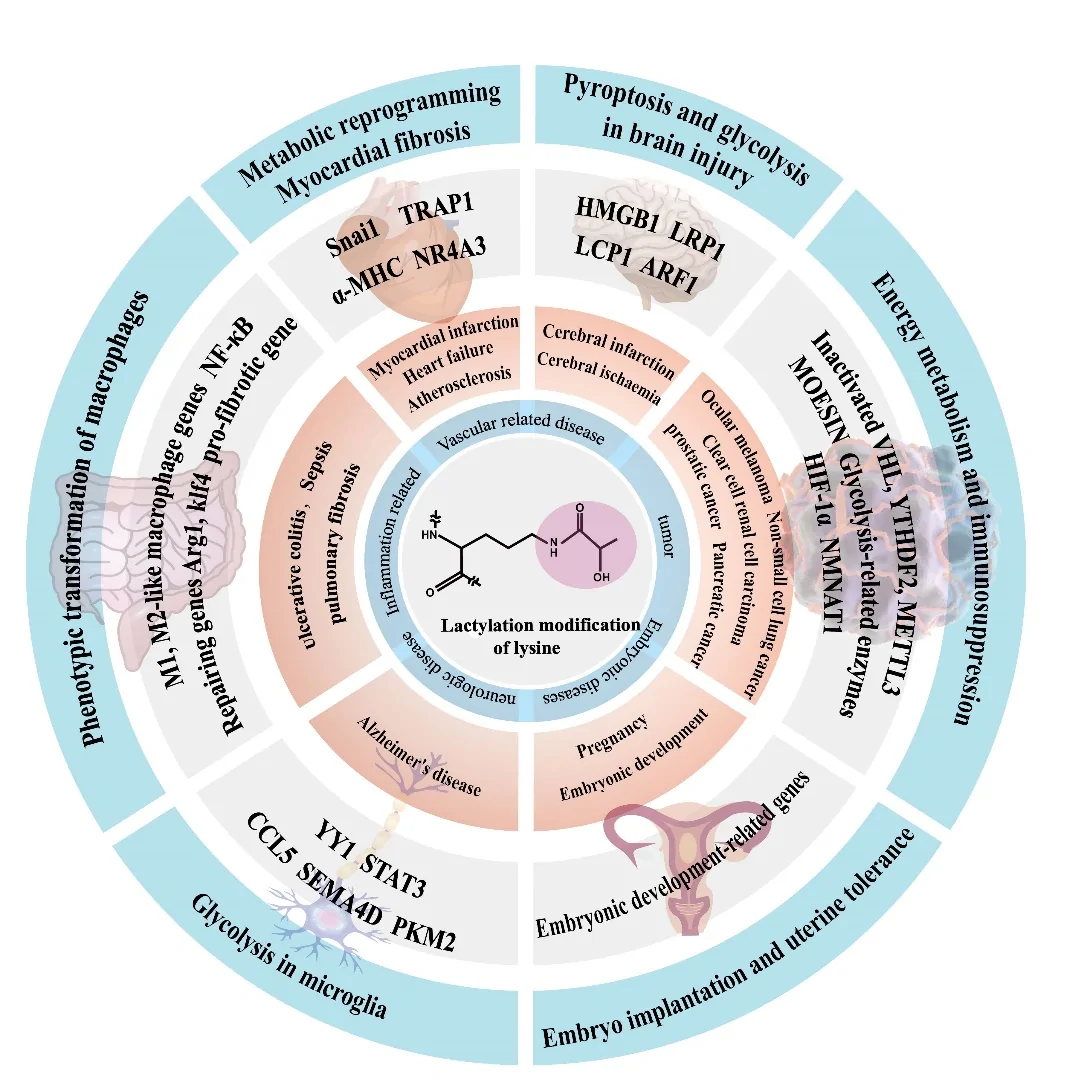
**This is a comprehensive diagram of lysine lactylation**. It focuses on the function of lysine lactonisation in the context of existing research. From the inside out: disease types, specific diseases, epigenetic regulatory genes, functional phenotypes. Snail1: TGF-β transcription factor; α-MHC: α-myosin heavy chain; HMGB1: High Mobility Group protein B1; LCP1: Lymphocyte cytosolic protein 1; YTHDF2: YTH N6-methyladenosine RNA-binding protein 2; METTL3: methyltransferase-like protein 3; PKM2: pyruvate kinase M2; VHL: von hippel-lindau arg1 arginase 1; Klf4: kruppel transcription factor family proteins; NR4A3: nuclear receptor subfamily 4 group A member 3; TRAP1: tumour necrosis factor receptor-associated protein 1; LRP1: Low-density lipoprotein receptor-related protein-1; ARF1: ADP-ribosylation factor 1; YY1: Yin Yang-1; STAT3: signal transducer and activator of transcription 3; CCL5: C-C motif chemokine 5; SEMA4D: Semaphorin4D; HIF1α: Hypoxia-inducible factor 1 subunit alpha; NMNAT1: Nicotinamide mononucleotide adenylyltransferease 1.

**Figure 3.**
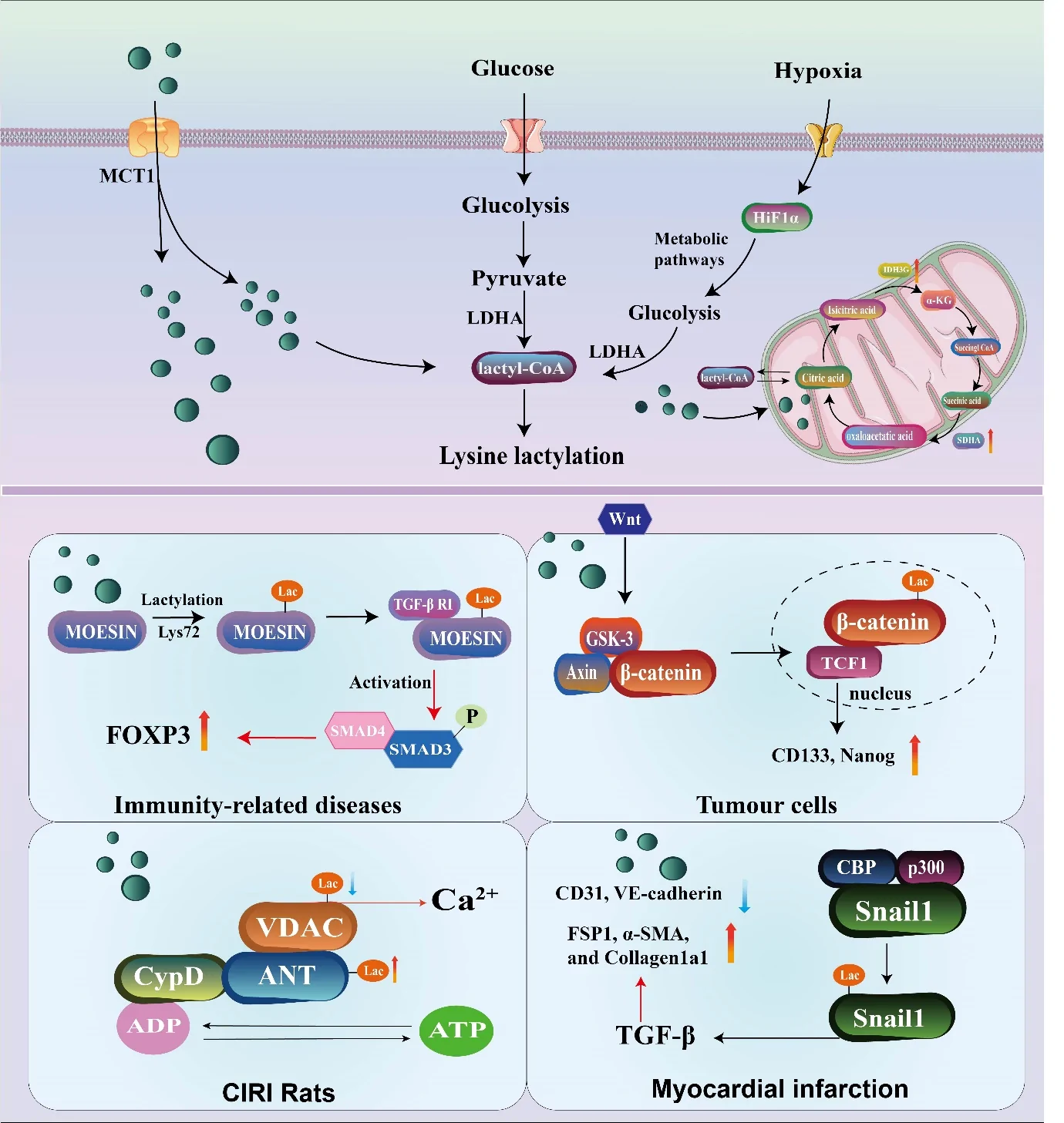
**Mechanisms of lactylation generation and associated signaling pathways**. Lactate is not only transported into cells via MCT1, but also produced from glucose via glycolysis. Lactate is even produced under hypoxic conditions, stimulating glycolysis and LDHA via HIF-1α in the metabolic pathway. More notably, lactate is able to synthesise lactoyl coenzyme A lactoyl transfer via acetyltransferase p300 and by histone deacetylases HDACs involved in histones and non-histone proteins, leading to a wide range of physiopathological activities in different diseases. In immune-related diseases lactate promotes tumourigenesis by regulating MOESIN lactylation and enhancing the TGF-β signalling pathway in regulatory T cells. Simultaneous lactylation of Snail1, a transcription factor of TGF-β, leads to endothelial-to-mesenchymal transition. β-catenin lactylation in the Wnt pathway promotes tumour cell growth and stemness. Lactylation of key proteins of the Ca2+ signalling pathway was detected in CIRI rats. MCT1: monocarboxylate transporters 1; LDHA: lactate dehydrogenase A; HIF-1α: hypoxia inducible factor 1 α; IDH: Isocltrate dehydrogenase; α-KG: α-ketoglutaric acid; SDHA: succinodehydrogenase; TGF-β R1: transforming growth factor β receptor1; FOXP3: forkhead box P3; GSK-3: glycogen synthase kinase 3; VDCA: voltage-dependent anion selective channel; ANT: adenine nucleotide translocase; CypD: cyclophilin D; Fsp1: fibroblast specific protein 1; α-SMA: α-smooth muscle actin.

Lactylation of both histones and non-histone proteins plays a pivotal role in the regulation of cellular functions associated with glycolysis, macrophage phenotypic shifts, vascular integrity, mitochondrial function, and neurological regulation (Table 1)[19, 21, 22, 25, 32-35]. These regulatory mechanisms are crucial for modulating neoplastic growth, invasiveness, and metastatic behavior, orchestrating inflammatory response and tissue repair, enhancing embryonic development, and attenuating the progression of cardiac failure and myocardial infarction [18, 22, 25, 27, 32, 33, 36-42] (Fig. 2). The influence of lactylation on the pathogenesis of diseases is intermediated by metabolic pathways involving glycolysis, the conversion of pyruvate, and the generation of lactate (Fig. 3) [19, 25, 29, 36]. The current understanding of the regulatory mechanisms by histone or non-histone lactylation is intricate and warrants additional investigative scrutiny.

Subsequent to conducting an in vitro cell-free analysis, it was established that histone lysine residue Kla undergoes lactylation through the catalytic action of the p300 enzyme. This process requires the presence of the p53 protein and is corroborated by additional research conducted on neoplastic cell cultures [17, 36]. Moreover, the dynamic addition and excision of the lactyl group on histone Kla by enzymatic means was observed, thereby excluding the hypothesis of a non-enzymatic pathway for this modification.

Lactylation, akin to diverse acyl modifications, encompasses both enzymes that writer and eraser these functional groups (Table 2). It has been shown that the lactylation writers and eraser are acyltransferases and deacetylation modifying enzymes, respectively [17]. The research group led by Yingming Zhao pioneered the exploration into the regulatory dynamics of lactylation on histones by assessing the influence of 18 histone deacetylases (HDACs). Through meticulous delactylation scrutiny, it was established that HDAC1-3 and SIRT1-3 contribute to the reduction of histone lactylation. The employment of the pan-HDAC inhibitor trichostatin A (TSA) and the targeted blockade of HDAC1-3 by apicidin markedly attenuated lactylation [43]. Research has pinpointed the deacetylase enzyme SIRT3 as a pivotal factor in the suppression of hepatocellular carcinoma (HCC), acting through inhibition of the progression of this malignancy by targeting the cell cycle regulator cyclin E2 (CCNE2) [33]. SIRT3 regulates lactylation on the K20 residue of the non-histone mitochondrial fission protein Fis1 by modulating high acetylation levels and the activity of Pyruvate dehydrogenase E1 component subunit alpha (PDHA1) [44]. Moreover, SIRT1 has been discovered to possess potent deacetylation capabilities in the context of cardiac failure, notably diminishing lactylation at lysine 189 of α-myosin heavy chain (α-MHC), a crucial modulator of cardiac insufficiency [25]. AGK2, a SIRT2 inhibitor, significantly enhances lactylation of methyltransferase-like protein 16 (METTL16) and m^6^A modification of ferredoxin 1 (FDX1) to induce copper-mediated cell death [34]. Zhang *et al*. have previously elucidated that lactylation of histones H3 and H4, a process contingent upon p53 and mediated by the acetyltransferase p300, exerts influence on gene transcription regulation [17]. Additionally, the modulation of high mobility group box 1 (HMGB1) lactylation by p300/CBP has been implicated in the pathogenesis of sepsis [37]. Furthermore, this post-translational modification is instrumental in facilitating the endothelial-to-mesenchymal transition through Snail1 lactylation, augmenting fibroblast growth factor (FGF) transcription by lactylated Yin Yang-1 (YY1), and intensifying retinal neovascularization [20, 35]. Consequently, HDAC1-3 and SIRT1-3 was definitively characterized as a robust lactylation eraser. Nonetheless, the implications of lactylation and its catalyzing enzymes in diverse pathological states warrant further elucidation.

**Table 2:** The different types of drugs targeting lactylation modifications are summarized.

Class	Compound	Target	IC50 EC50/μM	Mechanism	Effect	Ref.
**Acetyltransferase inhibitor**	C646	p300/CBP	0.4	Competitive inhibitor	Reduces HMBG1 lactylation levels	[37]
	A-485	p300	0.06	Competitive inhibitor	Inhibits transcriptional regulation and YY1 lactylation	[35]
	C646	p300	0.4	Competitive inhibitor	Suppressesα-MHC K1897 lactylation	[25]
**Acetyltransferase activator**	CTB	p300	85.43	Catalytic p300 enzyme activity	Promotes α-MHC K1897 lactylation	[25]
**Deacetylase inhibitor**	EX527	SIRT1	0.098	Competitive inhibitor	Increases α-MHC K1897 lactylation	[25]
	3-TYP	SIRT3	0.016	Competitive inhibitor	Facilitates lactate production and Fis1 K20 lactylation	[44]
**Deacetylase activator**	SRT1720	SIRT1	0.16	Catalytic SIRT1 enzyme activity	Reduces α-MHC K1897 lactylation	[25]
	AGK2	SIRT2	3.5	Catalytic SIRT2 enzyme activity	Increases in METTL16-K229 lactylation	[34]
	Honokiol	SIRT3	NA	Catalytic SIRT3 enzyme activity	Reduced CCNE2 lactylation.induced apoptosis	[33]

HMGB1: high mobility group box 1; YY1: Yin Yang-1; α-MHC: α-myosin heavy chain; Fis1: mitochondrial fission 1 protein; METTL16: methyltransferase-like protein 16; CCNE2: cyclin E2

## 3. Lactylation and Disease

Lactate pivotal function in myriad pathologies has been spotlighted, with its regulatory mechanisms emerging as a focal point in contemporary research. Accumulating data posits lactate as a biochemical precursor that facilitates lactylation, a process increasingly linked to a spectrum of illnesses including oncogenesis, cardiovascular anomalies, and neurodegenerative conditions. Lactylation modifications bear the capacity to modulate protein functionality and stability, thus playing a critical role in the etiology and advancement of various diseases. Investigations into the alterations of lactylation offer fresh insights into the etiopathogenesis of these conditions, concurrently spawning innovative approaches for their prognostic assessment and therapeutic management (Table 1). An in-depth comprehension of lactylation regulation mechanism is therefore of paramount importance in the realm of medical research, promising to yield novel biomarkers and therapeutic avenues for the precocious identification and intervention of diseases (Fig. 3). Lactylation modification is the alteration of protein function through the binding of lacate molecules to lysine residues of proteins, which is associated with cellular metabolism, inflammatory responses, and other physiopathological processes. Firstly, protein lactylation may affect the activity of transcription factors, which in turn elevates or decreases the level of Inflammation-related gene expression. Secondly, it can also act as a regulator of signal transduction and as a switch for activation or inhibition of signaling pathways. Finally, lactylation of proteins can change the original structure and charge properties of proteins, affecting the binding of proteins to downstream genes and so on. In conclusion, lactylation is an important post-translational modification that can achieve alterations in cellular signaling pathways in several of the ways described above, thereby affecting the course of disease [45].

### 3.1 Lactylation and inflammatory immune diseases

Historically regarded as merely a metabolic byproduct, lactate is now recognized for its critical involvement in immunomodulation. Current research indicates that lactate functions as a modulatory agent, possessing the capacity to either attenuate or amplify inflammatory responses, thereby playing a pivotal role in the homeostasis of both acute and chronic inflammatory processes [16]. Zhang *et al.* were pioneers in detecting lysine lactylation on lactate-induced histones via mass spectrometry analysis [17]. This discovery underscores lysine lactylation potential as an innovative mechanism to further elucidate influence of lactate on immune regulation, thus presenting a new avenue for investigating lactate role in immunomodulatory processes [17]. Lactylation modifications are closely related to inflammation and play an important role in regulating the inflammatory response. In some cases, lactate can promote the expression of inflammatory factors, thereby exacerbating the inflammatory response [46]. In addition, lactylation assists in the control and mitigation of inflammation by being able to repair the expression of repair and anti-inflammatory genes [38]. Next, we provide a detailed review of the role of lactation modifications in chronic versus acute inflammation, as well as potential therapeutic targets.

#### 3.1.1 Chronic inflammation and pulmonary fibrosis

It has been documented that the process of lysine lactylation modulates macrophage differentiation and activation, as noted in the literature [17]. Macrophages in an activated state play a pivotal role in the pathogenesis of ulcerative colitis. Using a macrophage-specific BCAP-knockout (BCAPΔMΦ) mouse model, the researchers observed elevated levels of pro-inflammatory mediators through ELISA, while western blot analysis showed reduced expression of the repair-associated genes Arg1 and Klf4. These changes were accompanied by dysregulated glycolytic pathways and lactate accumulation in BCAP-deficient miceBy generating a macrophage-specific BCAP-knockout (BCAPΔMΦ) mouse model using ELISA, the researchers observed an elevation in pro-inflammatory mediators, while western blot analysis revealed a reduction in the expression of repair-associated genes Arg1 and Klf4, concurrent with dysregulation in glycolytic pathways and lactate accumulation in BCAP-deficient mice. This dysregulation leads to decreased histone lactylation, subsequently reducing the expression of repair genes. However, the suppressed expression of these genes, a consequence of compromised histone lactylation and BCAP deficiency, can be ameliorated by the supplementation of exogenous lactate [32]. Hence, BCAP influences macrophage reparative transformation by modulating lactate amounts derived from glycolysis and dictating histone lactylation alterations, thereby affecting the transcription of genes implicated in tissue repair. Investigations revealed that lactate suppresses IL-1β synthesis, M1 macrophage polarization, NLRP3 inflammasome activation, and attenuates gut inflammation. Lactate also markedly enhances acetylation at histone H3K9 and lactylation at histone H3K18 within macrophages, prevents macrophage pyroptosis, and reinstates intestinal immune homeostasis [38]. Moreover, lactate induces metabolic and epigenetic reprogramming in pro-inflammatory Th17 cells, resulting in a marked reduction of IL-17A production and an indirect increase in Foxp3 transcription, accompanied by elevated histone H3K18la levels. Administering exogenous lactate to mice with intestinal inflammation markedly curtailed TH17 cell pathogenicity. These findings indicate that lactate-driven lactylation modifications denote that lactate orchestrates the epigenetic landscape of T cells and is crucial in the transformation of pro-inflammatory T cells into regulatory T cells [47].

Activated lung fibroblasts secrete lactate, which has been shown to facilitate the differentiation of myofibroblasts, an effect referred to as non-cell-autonomous action [48, 49]. Cui *et al*. have elucidated both intrinsic and extrinsic functions of aberrant glycolysis in pulmonary fibrosis [39]. Lactate, a byproduct of glycolysis, not only propels the fibrotic phenotype in macrophages but also influences the fibrogenic activities of alveolar macrophages. In addition, elevated levels of histone lactylation have been observed in a bleomycin and TGF-β1-induced experimental model of pulmonary fibrosis, which fosters the transcription of fibrogenic genes such as RG1, PDGFA, THBS1, and VEGFA in alveolar macrophages [39]. In this study, the transcriptional regulation of fibrosis genes was influenced by inducing histone lactylation modifications that impact the inflammatory response. In essence, lactate generated through glycolytic processes elicits lactylation in fibrotic pulmonary tissue, further promoting the expression of pro-fibrotic genes within alveolar macrophages. Ongoing studies are required to elucidate the impact of this epigenetic modification on additional cellular entities within the context of pulmonary fibrosis.

Altered microglial function and increased YY1 lactylation in autoimmune uveitis were identified through lactate content detection, pan-lactylation modifications, YY1 lactylation histology, and immunofluorescence. To elucidate the link between YY1 lactylation and microglial function, the study involved modulating lactate production, transfecting a YY1 K183 mutant virus, and adjusting p300, the primary lactylation acyltransferase, to influence YY1 lactylation. Using CUT & Tag technology, the researchers identified significant upregulation of inflammation-related genes associated with YY1 and confirmed the differential expression of these genes at transcriptional and translational levels [50]. In conclusion, the study demonstrates that retinal inflammation-induced lactylation accumulation promotes inflammation by modifying the lactylation of the key transcription factor YY1 (K183), which directly binds and regulates transcription of critical inflammatory genes, suggesting new avenues for uveitis diagnosis and treatment.

#### 3.1.2 Acute inflammation

Lactylation is implicated in acute inflammatory processes, and it is of particular note that elevated serum lactate concentrations correlate strongly with increased mortality rates in sepsis patients. Administration of lactate via peritoneal injection in septic murine models resulted in heightened serum lactate and HMGB1 levels markers inversely associated with murine survival in the context of lactate and HMGB1 expression. Furthermore, p300/CBP acetyltransferase has been validated as a critical enzyme that facilitating the lactylation of HMGB1 in macrophages. Lactate serves as a direct inducer of HMGB1 lactylation via the p300/CBP pathway. This lactylated/acetylated HMGB1 translocates from the nucleus to the cytoplasm and subsequently is excreted into circulation through exosomes. The release of exosomal HMGB1 contributes to endothelial cell integrity disruption and escalates vascular permeability [37]. Thus, targeting lactate metabolism and its downstream signaling could serve as a therapeutic strategy to mitigate HMGB1 release from macrophages and enhance survival probabilities in polymicrobial sepsis. Additionally, elevated lactylation levels and the expression of H3K18la were notably higher in septic shock patients compared to healthy controls, with H3K18la expression peaking in those with septic shock. The expression levels of pro-inflammatory cytokine IL-6 and anti-inflammatory cytokine IL-10 were also elevated, with H3K18la demonstrating a positive correlation with pro-inflammatory cytokines. In this research, H3K18la appears to exert an anti-inflammatory role, modulating the expression of genes involved in repair processes in septic patients [51, 52]. The acetylation of PDHA1 enhances lactate accumulation, leading to the modulation of Fis1 K20 lactylation modification via SAKI-mediated mitochondrial fission [44].

Moreover, evidence points to pyruvate kinase M2 (PKM2) as a pivotal regulator of the Warburg effect [53-56]. Consequently, Liu *et al*. indicated that inhibiting the PKM2 enzyme elevated glycolysis rates in macrophages, thereby amplifying the Warburg effect. External lactate enhances PKM2 activity in both murine models and macrophages, leading to increased Arg1 expression and decreased iNOS levels. These effects are reversed with the use of a PKM2 inhibitor, suggesting that lactate influences macrophage reparative phenotype conversion and accelerates healing by regulating PKM2 activity. Additionally, lactate seems to facilitate the LPS-induced transition of macrophages to a reparative state through the lactylation of PKM2 at the K62 site, thereby affecting wound healing [22]. Researchers observed a significant upregulation in lactylation levels following LPS treatment in sepsis-associated acute kidney injury (SA-AKI) mouse models and renal proximal tubular epithelial cells. Protein blotting analysis using a pan antibody to lactylation modifications confirmed the increased lactylation in SA-AKI models [57]. Mechanistically, histone lactylation exacerbates renal dysfunction by promoting inflammation, oxidative stress, and apoptosis through the RhoA/ROCK/Ezrin axis. Further studies comparing lactylation modifications in renal tissues from the cecum ligation and puncture (CLP) group with the sham-operated group revealed a significant upregulation in Ezrin lactylation, specifically at the K263 site, which was crucial in SA-AKI inflammation metabolism, as confirmed by Co-IP experiments. Elevated Ezrin-K263la levels activated the NF-κB signaling pathway, enhancing the inflammatory response and apoptosis when induced by LPS and lactic acid, whereas Ezrin-K263R mitigated HK-2 cell injury progression [57]. In other words, Ezrin-K263la promotes the progression of SA-AKI disease course

In conclusion, the inflammatory cascade induces lactate production and accumulation, which in turn may perturb lactylation modifications. These modifications play a role in the modulation of the immune response by altering the activity and functionality of immune cells, as well as by managing inflammatory signaling pathways. Lactylation not only has the potential to augment immune cell activity and decelerate disease progression, but also to regulate the magnitude and persistence of the inflammatory response by dampening immune cell function. Ultimately, lactylation is a significant contributor to both acute and chronic inflammatory states, exerting various regulatory effects across different disease spectrums.

### 3.2 Lactylation and tumours

#### 3.2.1 Energy metabolism in tumors

Despite the presence of oxygen, cancer cells exhibit a preference for glycolysis, leading to increased lactate production and metabolism compared to healthy cells [58]. This phenomenon suggests a direct impact of histone lactylation on cancerous cells. In ocular melanoma, Yu *et al*. identified an inverse relationship between histone lactylation and patient prognosis through immunoprecipitation and additional experimental methods. [36]. Targeting histone lactylation in these cells by reducing lactate with glycolysis inhibitors and lactate dehydrogenase-targeting siRNA further established the connection between lactylation and the malignancy. Histone lactylation has also been identified as a modifier of gene function, specifically influencing PER1/TP53 activity and cancer progression via the m6A reader protein YTHDF2. However, elevated lactylation levels in non-malignant cells result in minimal tumorigenesis, indicating it does not act as an initiating factor in tumor development. In non-small cell lung cancer, lactate treatment has been shown to alter metabolic enzyme expression, increasing tricarboxylic acid cycle enzymes (SDHA and IDH3G) and decreasing glycolytic enzymes (HK-1 and PKM), highlighting lactate role in metabolic gene expression changes and cancer progression [24].

Similarly, in clear cell renal cell carcinoma (ccRCC), deactivated von Hippel-Lindau (VHL) protein is associated with higher lactylation levels compared to normal tissue and has been linked to histone lactylation and the progression of ccRCC. Higher levels of histone lactylation lead to VHL inactivation. This inactivation of VHL leads to the upregulation of platelet-derived growth factor receptor β (PDGFRβ), which promotes ccRCC progression and further enhances histone lactylation, creating a self-amplifying cycle of malignancy [40]. The investigators found that inactive VHL activates transcription of PDGFRβ through active departure histone lactylation, which in turn promotes the progression of ccRCC. Likewise, PDGFRβ signaling can further stimulate histone lactylation, creating an oncogenic positive feedback loop in ccRCC. The Warburg effect driving metabolic reprogramming of stem cells is closely implicated in the treatment of certain cancers. Pan *et al*. found that the triterpene antitumor drug demethylzeylasteral (DML) inhibited the progression of hepatocellular carcinoma, and that the glycolytic metabolic pathway favors an antitumor effect.

Further studies revealed that the level of lactylation in hepatocellular carcinoma was noticeably higher than that in paracancerous tissues [59]. DML was able to inhibit the modification level of protein sites H3K9la and H3K56la by decreasing the intracellular lactate content of LCSCs, which resulted in the inhibition of histone lactate modification, thus suppressing the tumourigenicity of LCSCs. It is worth noting that this study mainly revealed the roles and mechanisms of glucose metabolism and epigenetic modification of LCSCs in the regulation of cellular function and the pathological process of hepatocellular carcinoma. Additionally, low SIRT3 expression in hepatocellular carcinoma tissues has been associated with advanced disease and poorer prognosis, with an inverse relationship between SIRT3 levels and lactylation. Studies have identified SIRT3-regulated lactylation modification proteins and sites, indicating that reduced SIRT3 levels facilitate cancer progression by enhancing lactate modifications of essential cell cycle proteins [33]. It provides novel perspectives for targeting cancer immunity by revealing the specific mechanism of Treg cell regulation by lactate. Researchers collected tumours and adjacent liver tissues from hepatitis B virus-associated hepatocellular carcinoma patients for lactomics and proteomics analyses to identify lactylation sites and proteins, found that lactylation mainly affects the metabolic pathways of carbohydrates, tricarboxylic acids, and amino acids [60]. The extensive and critical regulatory role in HCC cell metabolism was experimentally validated by the ability of Kla to regulate the function of metabolism-associated proteins and potentially facilitate the progression of HCC. Energy metabolism dysregulation is a key feature of rapid tumor cell growth, and HPV enhances ATP production via aerobic glycolysis, contributing to cervical cancer development [61, 62]. Studies have shown that pentose phosphate pathway (PPP) metabolites are markedly elevated in cells with ectopic expression of HPV16 E6E7 [63]. The PPP is activated to manage oxidative stress and maintain intracellular redox balance [62]. Lactylation at the K45 site of glucose-6-phosphate dehydrogenase (G6PD), the rate-limiting enzyme of PPP, inhibits G6PD activity. However, HPV16 E6 prevents this lactylation modification, promoting G6PD dimer formation, which enhances its oncogenic effects. The lactylation of G6PD leads to a modification in its structure and consequently its function [63]. This discovery could offer an effective strategy for cervical cancer treatment. Lactate enhances the survival of pancreatic adenocarcinoma (PAAD) cells during glucose deprivation, primarily through mitochondrial respiration driven by GLS1-mediated glutamine catabolism. Metabolomic analyses and various experimental validations have shown that lactylation activates the NMNAT1-mediated NAD salvage pathway, preventing NAD depletion caused by glucose deprivation. Elevated lactylation levels in PAAD patients are closely associated with poor prognosis. Importantly, NMNAT1 lactylation plays a crucial role in sustaining Sirt1 activity and promoting PAAD cell survival [64]. Consequently, targeting both lactate and NAD metabolism may represent a promising therapeutic strategy for PAAD treatment.

#### 3.2.2 Immune response in Tumors

Moreover, research by Wang *et al*. demonstrated an elevation in methyltransferase-like protein 3 (METTL3) levels within the tumor-infiltrating myeloid cells (TIMs) extracted from colon carcinoma patient biopsies, an observation that was associated with a poor clinical outcome [19]. Subsequent research demonstrated that lactate promotes METTL3 transcription in these TIMs via histone H3K18 lactylation. This post-translational modification is capable of occurring at the CCCH-type zinc finger motif within the METTL3 enzyme, which is pivotal for RNA recognition and subsequent m6A RNA methylation activity. At the intersection of metabolism, epigenetics, and transcription, this lactylation-driven alteration governs the immunosuppressive capabilities of TIMs, ultimately facilitating immune evasion by neoplastic cells. Compelling data implicates the gut microbiota in modulating the pathogenesis of various disorders, ranging from obesity and inflammatory bowel disease to metabolic syndrome and neoplasia [65-68]. In the context of colorectal cancer, Wang *et al*. effectively promoted the up-regulation of LNC000152 through the application of LPS, which is implicated in gastrointestinal inflammation as well as neoplastic cell migration and invasion, the latter being facilitated by histone lactylation [69]. These findings underscore the significance of lactylation in gastrointestinal malignancies and herald new therapeutic avenues for these conditions. In the realm of immunoregulation, Treg cells are known to mitigate autoimmunity and sustain immunological equilibrium [70]. However, lactate is found to bolster Treg cell stability and functionality by facilitating the lactylation of the cytoskeletal protein MOESIN at Lys72. This modification influences MOESIN's interaction with the TGF-β receptor I and the downstream SMAD3 pathway, thereby modulating Treg cell lineage commitment (Fig. 3) [27]. The lactylation modification of MOESIN enhances its stability and regulates the downstream SMAD3 signaling pathway.

In addition, the upregulation of β-catenin in hypoxic environments is implicated in the proliferation of colorectal neoplasms by stimulating the Wnt/β-catenin signaling cascade, influencing the preservation of cellular pluripotency attributes, thereby altering neoplastic progression [23]. Lactate-induced lactylation of METTL16 at lysine 229 promotes m6A modification of FDX1 mRNA, resulting in increased FDX1 mRNA expression and subsequent copper-dependent cell death in gastric cancer [34]. Similarly, Zhang *et al.* have confirmed lactylation in gastric cancer through proteomic analysis and immunoblotting techniques, yet the precise modulatory mechanisms and their impact on neoplastic progression remain unexplored [71]. Ducts contribute to the malignant characteristics of gastric cancer, influencing its progression and prognosis. Luminal HIF-1α inhibits immune surveillance and drives luminal plasticity, leading to impaired signaling. Research indicates that the nuclear protein HIF-1α can undergo lactylation. In prostate cancer, elevated lactate uptake via MCT1 results in the lactylation of HIF-1α, which allows it to maintain high stability in normoxic environments. Furthermore, increased levels of HIF-1α promote the expression of KIAA1199, while KIAA1199 enhances its transcriptional activity through the activation of associated signaling pathways. In prostate cancer tissues, KIAA1199 expression is notably elevated, further facilitating angiogenesis and the advancement of prostate cancer [72].

Collectively, lactylation modifications possess the capacity to modulate immune cell functions, thereby altering the neoplastic microenvironment, facilitating immune evasion and infiltration by neoplastic cells, and promoting the upregulation of oncogenic gene expression. Concurrently, these modifications exert direct control over the metabolic enzymes of neoplastic cells, mediating tumoral metabolism, and influence neoplastic development via signaling pathways and other mechanisms.

### 3.3 Lactylation and cardiovascular diseases

#### 3.3.1 Lactylation in brain diseases

Cerebrovascular ischemia-reperfusion (CI/R) has been implicated in the initiation of diverse cellular demise mechanisms including necrosis, apoptosis, and pyroptosis [73-75]. Recent research has homed in on the investigation of localized cellular death within this context [76]. HMGB1 has been previously identified as a promoter of focal cell death in disparate conditions, such as Kawasaki disease [77]. Research by Yao *et al*. has revealed an upregulation of HMGB1 during CI/R, concomitant with significant increments in lactate dehydrogenase A (LDHA) activity, lactate accumulation, and H3K18 lactylation within the promoter vicinity of the HMGB1 gene. Silencing LDHA was shown to diminish H3K18 lactylation, a process reversible through exogenous lactate supplementation. This suggests a mechanism where LDHA-driven histone lactylation modulates HMGB1 activity, thereby contributing to focal cell death in CI/R scenarios [41]. Additional investigations into cerebral ischemic events have indicated a marked escalation in infarction dimensions in a CIRI rat model compared to controls, with a noticeable elevation in protein lactylation within the cerebral endothelium, as identified by pan-anti-Kla antibody marking. This finding was corroborated by 4D label-free quantitative proteomic analysis, which focused on lactylation-specific alterations. Pathway analysis via KEGG highlighted a predominance of Ca^2+^ and MAPK signalling pathways in CIRI, with critical proteins within the Ca^2+^ pathway-specifically SLC25A4 (K245) and SLC25A5 (K96)-undergoing lactate modifications (Fig. 3) [26]. Zhang *et al*. further established through Western blotting and co-immunoprecipitation assays that lactylation of the leukocyte cytosolic protein 1 (LCP1) was augmented in both MCAO rats and OGD/R-treated cells, with a more pronounced effect post-supplementation of exogenous lactate. However, this lactylation was attenuated when glycolysis was inhibited by 2-deoxy-D-glucose (2-DG) [42, 78]. Briefly, the modulation of LCP1 lactylation appears to play a pivotal role in the pathophysiology of CI. Ischemic stroke results in dysfunction of mitochondrial oxidative phosphorylation and stress, with low-density lipoprotein receptor-related protein-1 (LRP1) implicated in this condition [79-81]. Previous studies have shown that LRP1 plays a role in regulating metabolic homeostasis in the central nervous system and glucose uptake in astrocytes, influencing lactate production [82-84]. Further research has identified that LRP1-regulated lactate metabolism impacts mitochondrial efflux in astrocytes, demonstrating that the lactylation modification at the ADP-ribosylation factor 1 (ARF1) K73 site is involved in this regulation [85]. Reduced lactylation at ARF1 K73 modulates the transfer of healthy mitochondria from astrocytes to neurons, potentially mitigating neurological damage following cerebral ischemia

#### 3.3.2 Lactylation in the cardiovascular

Lactylation exerts a significant influence on both neuronal and cardiovascular tissues. Empirical evidence strongly associates elevated lactylation with the pathogenesis of heart failure [86, 87]. The inflammatory cascade and subsequent biological processes are critical for myocardial repair post-infarction [88]. However, an overactive inflammatory response can exacerbate myocardial injury and compromise cardiac function [89, 90]. Wang *et al*. highlighted a surge in lactylation and H3K18la modification in monocyte-macrophages within 4-24 hours post-myocardial infarction. Further studies demonstrated that H3K18la plays a role in activating reparative gene expression, including Lrg1, Vegf-a, and IL-10, thereby improving myocardial function in mouse models. These investigations revealed a metabolic transition in monocytes from oxidative phosphorylation to glycolysis post-myocardial injury, affecting lactylation and gene expression linked to this epigenetic modification. The involvement of GCN5 as a lactylation agent and the regulatory mechanism of GCN5-mediated H3K18la via the IL-1β/IL-1R1 axis were substantiated through molecular docking, co-immunoprecipitation, and RNAi methodologies [91]. Additionally, post-infarction endothelial-to-mesenchymal transition has been linked to increased cardiac fibrosis and dysfunction, with TGF-β signaling and Snail1 expression being pivotal in this process [92-94]. Epigenetic alterations are recognized as modulators of gene expression during cardiac fibrogenesis [95]. Thus Fan *et al*. showed that administration of the glycolytic inhibitor 2-DG markedly enhanced cardiac function, attenuated myocardial fibrosis, and impeded EndoMT in affected mice. Lactate was shown to activate the TGF-β/Smad2 pathway and elevate Snail1 lactylation, which enhances its interaction with CBP/p300. In vivo experiments confirmed that downregulating Snail1 not only improved cardiac performance but also inhibited the EndoMT process in myocardial infarction models, underscoring the significant role of Snail1 lactylation in promoting EndoMT via the TGF-β/Smad2 axis (Fig. 3) [20].

Recent insights have revealed that diminished lactylation in heart failure exacerbates myocardial fibrosis and cardiac impairment. Novel findings identified Titin K1897la as crucial for Titin's binding to α-MHC, ensuring cardiac structural and functional integrity. Hypertension-induced myocardial damage was attributed to reduced Titin K1897la, disrupting the Titin-α-MHC complex and precipitating cardiac failure. Conversely, sodium lactate supplementation and MCT4 inhibitor VB124 therapy notably elevated Titin K1897la levels, mitigating myocardial damage and ameliorating heart failure [25]. This research synthesizes the central mechanisms of hypertensive end-organ damage and presents novel protective strategies, yet the intricate mechanisms of lactylation in cardiovascular pathology warrant further exploration.

Compelling evidence indicates a strong link between vascular calcification and histone lactylation. Ma *et al*. identified an upregulation of nuclear receptor 4A3 (NR4A3) in calcified vascular regions. Notably, NR4A3 enhances glycolysis and lactylation by binding to the promoter regions of the glycolytic genes ALDOA and PFKL, thereby promoting their transcription. Furthermore, H3K18la facilitates vascular calcification, while suppression of NR4A3 expression decreases H3K18la levels and mitigates vascular calcification. Furthermore, H3K18la facilitates vascular calcification, while suppression of NR4A3 expression decreases H3K18la levels and mitigates vascular calcification. Targeting NR4A3-mediated metabolome-epigenome signaling pathways could offer novel strategies for preventing vascular calcification [96]. Recent research indicates that elevated tumour necrosis factor receptor-associated protein 1 (TRAP1) expression reprograms energy metabolism, leading to senescence and atherosclerosis (AS) in vascular smooth muscle cells (VSMCs). The upregulation of TRAP1 enhances glycolysis, resulting in lactate buildup. Concurrently, it suppresses the expression of the novel histone delactylase HDAC3, facilitating the formation of H4K12la, which modifies the histone microenvironment and regulates the transcriptional activation of SASP (senescence-associated secretory phenotype). This process further contributes to VSMC senescence and AS. These findings offer new insights into the interplay between energy metabolism reprogramming and epigenetic modifications, revealing novel mechanisms underlying cellular senescence and AS [97].

### 3.4 Lactylation and Neuropsychiatric diseases

Elevations in cerebral lactate have been observed in a spectrum of neuropsychiatric conditions, notably major depressive disorder and panic disorder [98, 99]. Contemporary research elucidates that brain cells undergo lactylation, a process modulated by lactate concentration and neural activity [100]. In murine models, electroconvulsive stimulation triggers a rise in brain neuron excitation, concomitant with an increase in lactic acidosis. The induction of the social defeat stress (SDS) model for depression yielded a marked upsurge in the neurobiomarkers c-Fos, lactate, and lactylation levels, implying that stress-induced neural excitation may initiate lactylation, thus affecting emotional behaviors and establishing a connection between depression and protein lactylation induced by neural hyperactivity. Alzheimer's disease (AD), a prevalent neurodegenerative condition, is escalating in incidence, with emerging evidence suggesting a pivotal role of microglia in its etiology. Analysis of both clinical AD cerebral samples and transgenic mice with five familial Alzheimer’s disease brain specimens revealed hyperactive microglial glycolysis resulting in intracellular lactate accumulation and subsequent augmentation of Pan Kla and H4K12la in the AD cerebrum. The elevation in pan Kla and histone lactylation modifications triggers the expression of genes associated with glycolysis [18]. Consequently, a self-reinforcing "glycolysis-histone lactylation-PKM2" feedback loop is established, perturbing microglial glucose metabolism, disrupting homeostasis, inciting neuroinflammation, and facilitating AD progression. Thus, intervention targeting PKM2 to inhibit this cycle may reinstate microglial functionality, mitigate neuroinflammation, and ameliorate AD pathology. Despite these insights, lactylation role in neuropsychiatric disorders warrants further study to unravel underlying mechanisms.

### 3.5 Lactylation and embryonic development

Oxygen serves as a crucial determinant in the progression of preimplantation embryos, with its concentration pivotal in modulating the expression of transcription factors and genes implicated in the glycolytic cascade [101]. Hypoxia induces a decrement in glycolytic flux, culminating in diminished lactate synthesis [102]. Yang *et al*. have delineated a direct proportionality between embryonal lactate levels and histone lactylation within murine preimplantation embryos. Embryo culture under varying oxygen tensions, specifically atmospheric, physiological, gradient, and hypoxic conditions, demonstrated a marked diminution in H3K23la and H3K18la during the blastocyst stage in embryos subject to gradient and hypoxic environments. The enzyme lactate dehydrogenase A (LDHA) is responsible for the transformation of pyruvate into lactate amidst glycolysis [36]. A comparative analysis with embryos cultured under normoxia revealed that hypoxic conditions precipitated a reduction in LDHA expression, lactate generation, and histone lactylation, alongside a concurrent decline in the advancement of embryos to the blastocyst stage. This underscores the deleterious effects of hypoxic stress on the developmental capacity of preimplantation embryos.

Furthermore, the application of LDHA inhibitors has been shown to substantially attenuate LDHA activity, embryonic cellular lactate concentrations, and the initiation of lactylation, thereby impeding embryonic progression [103]. Using the ovine model, the ligand-receptor-pathway (LRP) method has revealed that during embryo attachment, the increase in lactate production through enhanced glycolysis acts as an endogenous signal to trigger uterine responses and strengthen H3K18la in the endometrium, thereby reshaping uterine receptivity [104]. Such insights unveil novel paradigms in embryogenesis and furnish a robust scientific rationale for enhancing embryonic development and successful pregnancy outcomes.

### 3.6 Lactylation and aging

Prior investigations have illuminated that microglial cells are capable of metabolic modulation from oxidative phosphorylation to glycolysis when activated by certain stimuli, a shift of critical significance as the resultant lactate facilitates the secretion of proinflammatory mediators via lactylation processes [18, 105, 106]. Convincing data substantiates the notion that the inflammation induced by aged microglia is a contributory factor in Alzheimer's disease pathogenesis. Research by Wei *et al.* has demonstrated a positive correlation between lactate concentrations and H3K18 lactylation in both senescent microglia and the hippocampal regions of aging and Alzheimer's afflicted murine models. The primary regulatory mechanism involves H3K18 lactylation modulating the senescence-associated secretory phenotype (SASP), specifically via the NF-κB pathway's induction of IL-6 and IL-8, thereby intensifying neuroinflammation, brain aging, and the progression of Alzheimer's disease [21]. These insights offer novel avenues for therapeutic intervention targeting cerebral senescence [21]. Contemporary studies have observed that senescent cells undergo a PDK4-dependent shift towards enhanced aerobic glycolysis and augmented lactate production, a metabolic reprogramming [107]. Pharmacological targeting of PDK4 has been shown to interrupt the senescence cascade within the neoplastic microenvironment. Moreover, lactate has been implicated in the elevation of reactive oxygen species through NOX1, fueling the SASP, while downregulation of PDK4 expression attenuates DNA damage and dampens the SASP [107]. Clinical observations have posited that PDK4 inhibitors may mitigate age-related physiological decline and forestall chronic age-associated diseases, suggesting a metabolic interconnection between cellular senescence, lactate production, and aging pathologies. Nevertheless, the putative association with lactylation remains an area ripe for further scientific scrutiny. Several studies have indicated a close connection between the metabolism of the nucleus pulposus (NP) and intervertebral disc degeneration (IDD) [108-110]. The primary metabolic pathway in NP cells is glycolysis, with lactic acid produced from glycolysis being a key contributor to IDD [111, 112]. Conversely, glutamine induces metabolic reprogramming [113]. Zhang *et al*. discovered that glutamine levels were reduced, while lactate and lactylation levels were elevated in NP tissues from severely degenerated human and rat samples. The underlying molecular mechanism involves glutamine inhibiting glycolytic metabolism, thereby decreasing lactate production. This reduction down-regulates AMPKα lactylation and activates phosphorylated AMPKα expression, ultimately delaying aging and promoting autophagy and matrix synthesis [114]. The process of aging is characterized by numerous protein modifications, with alterations in histone and non-histone lactylation levels potentially serving as biomarkers for aging and related diseases. These biomarkers are crucial for evaluating an individual health status and the aging process. Furthermore, lactylation has been implicated in neurodegenerative diseases and metabolic syndrome, offering potential new targets for anti-aging therapies [115, 116]. In conclusion, lactylation modifications are important in aging and are expected to provide new perspectives and ideas for probing aging mechanisms and developing therapeutic strategies.

## 4. Discussion

Lactate, a crucial byproduct of the glycolytic pathway, serves as both a metabolic feedback modulator and a signaling intermediary in the context of immunological dysfunctions and the neoplastic stroma, influencing oncogenesis by altering various cell signaling cascades [117]. This metabolite preserves the niche for neoplastic growth and fosters an immunosuppressive milieu that facilitates the evasion of tumor cells from immune detection. Additionally, lactate-mediated lactylation sustains the gene expression involved in neoplastic homeostasis. Consequently, targeting the regulatory mechanisms of lactate production or histone lactylation could represent a novel therapeutic strategy for malignancies. In inflammatory states, lactate role is multifaceted, it may aggravate arthritis by diminishing glycolysis and augmenting lipogenesis via the restriction of CD4^+^T lymphocyte motility, thus perpetuating inflammation [118]. Conversely, lactate can downregulate the synthesis of pro-inflammatory mediators in macrophages [38, 119, 120]. Complicating the external modulation of inflammation and necessitating further investigative efforts.

The phenomenon of cellular senescence has garnered considerable attention in scientific research, revealing that cells in the senescent state exhibit a distinctive metabolic signature. This signature is characterized by an upregulation of glucose transporters and enzymes involved in glycolysis, leading to an augmented glycolytic flux. Furthermore, there is an enhancement in the activities of the tricarboxylic acid (TCA) cycle and oxidative phosphorylation, culminating in an elevated production of lactate [107]. Recent investigations have elucidated the role of lactate as a modulatory agent in senescence, notably through its capacity to influence epigenetic alterations involving histone lactylation. This biochemical process has been implicated in the etiology of cerebral senescence and AD by mediating the H3K18la/NFκB signaling pathway within both naturally aged and AD-affected microglial cells, as well as within hippocampal tissue [21]. Further investigation is warranted into the relationship between histone lactylation and senescence, as well as its potential pivotal influence within highly glycolytic cellular populations such as astrocytes and leukocytes, as delineated in comprehensive reviews on lactylation. Histone lactylation is instrumental in cells with high glycolytic rates, such as astrocytes and leukocytes, and preliminary studies have implicated lactylation in embryonic development, myocardial infarction, and other pathologies and DNA damage [121-123]. In summation, lactate and its associated lactylation are potent regulatory elements across a spectrum of diseases, and interventions in this metabolic process may emerge as a critical therapeutic approach.

Within lactylation research, distinguishing the functional impacts of lysine residue modifications poses a recurrent challenge, as the effects of acetylation and lactylation are similar, and p300/CBP acetyltransferases frequently act as lactylation enzymes. This similarity complicates the determination of the specific outcomes attributable to lactylation. Histone acetylation modifies the electrostatic and spatial configuration of histones, diminishing their affinity for DNA and thus enhancing the accessibility of transcriptional activators [124]. Histone lactylation, on the other hand, exhibits distinct temporal patterns and regulatory influences on gene transcription [91]. Zhang *et al*. demonstrated that genes marked by increased histone lactylation are unique, with many not showing significant upticks in acetylation [17], indicating a superior role for lactylation in gene regulation. Given that both modifications occur on lysine residues, it is conceivable that some form of interplay or competition between them may exist, representing an important aspect of future lactylation research.

The modulation of gene expression by histone lactylation has been established, however, the broader implications of lactylation may lie in the realm of non-histone proteins. Gao *et al*. reported substantial lactylated proteins within the nuclear, mitochondrial, and cytoplasmic compartments of grey moulds, suggesting non-histone lactylation as a predominant form [28]. Investigations into non-histone lactylation across various pathologies including polymicrobial sepsis, neoplasms, inflammatory conditions, and cardiovascular diseases have provided ample evidence of its pervasive presence across human organ systems and its role in maintaining physiological homeostasis alongside other post-translational modifications [19, 20, 22, 23, 27, 37, 42, 60]. The function of non-histone protein lactylation has been partially elucidated, revealing its capacity to modulate protein interactions and downstream binding through lactylation [25]. Furthermore, lactylation may either promote protein degradation or contribute to protein stability [23, 42]. Unlike histone lactylation modification, non-histone lactylation modification has more diverse functions. It may not only regulate gene expression, but more importantly, it can act as a signaling molecule that interacts with multiple signaling pathways in the cell in response to external stimuli. By studying new protein modifications, we expect to be able to target the relevant enzyme or target of this modification to ameliorate human diseases. In addition, since lactylation is widespread in all cells in the body, enhancing or reducing lactylation modifications in relevant cells in rare diseases is faster and more promising than anchoring a gene. While the full scope of non-histone lactylation function remains to be uncovered, current research suggests its potential to precisely and directly influence cellular processes. Future investigations may focus on lactylation involvement in cellular processes such as the cell cycle, DNA repair mechanisms, and autophagy [124, 125].

In the realm of the current investigation, an array of pharmaceuticals aimed at the post-translational modification of proteins has been successfully deployed in clinical settings, notably in oncological therapeutics, where they have garnered impressive outcomes. The utilization of deacetylase inhibitors, including Vorinostat, Belinostat, Romidepsin, and Panobinostat, in the treatment of lymphoma and myeloma has been well-documented [126-129]. Vorinostat enhances histone acetylation by inhibiting HDAC family proteins, which in turn affects gene expression. Olaparib is an inhibitor of PARP that promotes tumor cell death by affecting post-translational modifications of proteins regulated by DNA repair processes. Furthermore, lactate has been posited as a novel oncological therapeutic target, with agents that inhibit lactate transporter proteins MCT1 and MCT4 presently in the preclinical and clinical trial phases [130, 131]. Notably, histone lactylation has been implicated in the pathogenesis of a multitude of diseases, with the inhibition of said lactylation poised to emerge as a pivotal therapeutic target for related pathologies in the future, including but not limited to the lactylation of H3K9 and H3K56 in LCSC 49, and H3K18 lactylation in ocular melanoma and colorectal carcinoma [19, 36].

Moreover, H3K18la has also been identified as a marker in inflammatory conditions, and indeed, inflammation is known to precipitate elevated serum lactate levels, which bear clinical significance for the diagnosis and can be quantified through serum lactate measurements [37]. In addition to inflammation, the assessment of lactate serum levels may provide insights into disease states, with therapeutic strategies aimed at reducing blood lactate levels potentially yielding beneficial effects. The role of non-histone lactylation in disease etiology, including MHC lactylation in cardiovascular pathology and β-catenin lactylation in neoplastic conditions, is also of significance. The enzymatic regulation of these lactylation processes by lactonase and delactonase, through specific activators or inhibitors, may hold considerable promise for clinical research [23, 25]. Furthermore, histone lactylation has been identified as an epigenetic hallmark of the glycolytic shift, exhibiting heightened sensitivity to lactate levels and thus presenting a novel therapeutic avenue [17]. Inhibition of the lactate-producing glycolytic pathway might also represent a potential therapeutic strategy for lactylation-targeted treatments.

Currently, the identification of lactate sites is mostly done by mass spectrometry, which has the advantages of high sensitivity and high resolution. In addition, metabolomics analysis is also a promising approach, and it is expected to be able to identify more cells in which lactylation plays an important function by studying metabolomics to analyze the levels of lactate and its metabolites in the relevant cells [132]. Metabolomics involves examining alterations in small molecule metabolites within the body triggered by various stimuli. By evaluating an individual lactate metabolic profile, it aids in crafting personalized therapeutic strategies and disease management plans, thereby enhancing disease screening and treatment. When integrated with transcriptomics and proteomics, metabolomics can help build gene regulatory networks and elucidate the molecular mechanisms underlying disease processes. Future advancements may see its integration with diverse genomics technologies to achieve multi-omics fusion, and it is anticipated to combine with systems biology methods for comprehensive metabolic network analysis, revealing the specific mechanisms of lactylation and other modifications in disease. As for the choice of therapeutic options, lactylation is usually regulated by genes for lactylation-modifying enzymes, such as LDHA [133, 134]. The development of activators or inhibitors that regulate such enzymes is expected to modulate lactylation modification levels in vivo. In addition, regulating cellular metabolism through the development of new small molecule drugs and developing new ways of gene editing have the potential to modulate the level of lactylation and therefore the disease process. Moreover, the advancement of novel small molecule therapeutics and innovative gene editing techniques to alter cellular metabolism, such as creating small molecule compounds that precisely identify and inhibit lysine lactylase activity, thereby reducing in vivo lactate levels, or targeting specific lactylation-related pathways, is anticipated to regulate lactylation modification levels in vivo and subsequently influence disease progression.

In conclusion, the elucidation of lactylation has significantly expanded the epigenetic landscape, offering novel therapeutic avenues for disease management. Nonetheless, a comprehensive understanding of the functional phenotypes and underlying mechanisms of lactylation remains imperative. Future research endeavors will also encompass the development of pharmaceuticals that not only exhibit fewer adverse effects compared to existing modalities but also boast enhanced disease-specific regulatory capabilities.

## 5. Conclusion

To sum up, lactate-induced lactylation serves as a critical modulator of macrophage polarization, sustains immunological equilibrium, and is implicated in pivotal biological processes including neuroregulation, angiogenesis, and T lymphocyte activation. The pathogenesis of several disorders, notably oncogenesis, ischemic encephalocardiovascular pathology, immune cell activation syndromes, neuropsychiatric conditions, embryogenesis, and cardiac insufficiency, has been intimately associated with lactylation pathways. The identification of this post-translational modification (PTM) of proteins heralds a novel research vista and heralds the advent of innovative therapeutic targets for a spectrum of diseases.
